# Protective Efficacy of Recombinant Turkey Herpes Virus (rHVT-H5) and Inactivated H5N1 Vaccines in Commercial Mulard Ducks against the Highly Pathogenic Avian Influenza (HPAI) H5N1 Clade 2.2.1 Virus

**DOI:** 10.1371/journal.pone.0156747

**Published:** 2016-06-15

**Authors:** Walid H. Kilany, Marwa Safwat, Samy M. Mohammed, Abdullah Salim, Folorunso Oludayo Fasina, Olubunmi G. Fasanmi, Azhar G. Shalaby, Gwenaelle Dauphin, Mohammed K. Hassan, Juan Lubroth, Yilma M. Jobre

**Affiliations:** 1 Reference Laboratory for Veterinary Quality Control on Poultry Production (NLQP), Animal Health Research Institute, P.O. Box, 264, Giza, Egypt; 2 Food and Agriculture Organization of the United Nations (FAO)–Emergency Center of Transboundary Animal Diseases (ECTAD), P.O. Box, 2223, Giza, Egypt; 3 Department of Veterinary Tropical Diseases, Faculty of Veterinary Science, University of Pretoria, Private Bag X04, Onderstepoort, South Africa; 4 Department of Production Animal Studies, Faculty of Veterinary Science, University of Pretoria, & Department of Animal Health, Federal College of Animal Health and Production Technology, Ibadan, Nigeria; 5 Food and Agriculture Organization (FAO) Viale delle Terme di Caracalla, 00153 Rome, Italy; The University of Chicago, UNITED STATES

## Abstract

In Egypt, ducks kept for commercial purposes constitute the second highest poultry population, at 150 million ducks/year. Hence, ducks play an important role in the introduction and transmission of avian influenza (AI) in the Egyptian poultry population. Attempts to control outbreaks include the use of vaccines, which have varying levels of efficacy and failure. To date, the effects of vaccine efficacy has rarely been determined in ducks. In this study, we evaluated the protective efficacy of a live recombinant vector vaccine based on a turkey Herpes Virus (HVT) expressing the H5 gene from a clade 2.2 H5N1 HPAIV strain (A/Swan/Hungary/499/2006) (rHVT-H5) and a bivalent inactivated H5N1 vaccine prepared from clade 2.2.1 and 2.2.1.1 H5N1 seeds in Mulard ducks. A 0.3ml/dose subcutaneous injection of rHVT-H5 vaccine was administered to one-day-old ducklings (D1) and another 0.5ml/dose subcutaneous injection of the inactivated MEFLUVAC was administered at 7 days (D7). Four separate challenge experiments were conducted at Days 21, 28, 35 and 42, in which all the vaccinated ducks were challenged with 10^6^EID_50_/duck of H5N1 HPAI virus (A/chicken/Egypt/128s/2012(H5N1) (clade 2.2.1) via intranasal inoculation. Maternal-derived antibody regression and post-vaccination antibody immune responses were monitored weekly. Ducks vaccinated at 21, 28, 35 and 42 days with the rHVT-H5 and MEFLUVAC vaccines were protected against mortality (80%, 80%, 90% and 90%) and (50%, 70%, 80% and 90%) respectively, against challenges with the H5N1 HPAI virus. The amount of viral shedding and shedding rates were lower in the rHVT-H5 vaccine groups than in the MEFLUVAC groups only in the first two challenge experiments. However, the non-vaccinated groups shed significantly more of the virus than the vaccinated groups. Both rHVT-H5 and MEFLUVAC provide early protection, and rHVT-H5 vaccine in particular provides protection against HPAI challenge.

## Introduction

Egypt is one of five countries where the highly pathogenic avian influenza (HPAI) H5N1 has become endemic. The H5N1 virus continues to circulate and has already caused great economic losses in poultry [1, 2]. Human infections and deaths have also been reported. Approximately 100 to 150 million domestic ducks are produced in Egypt annually and this makes ducks the species that constitutes the second largest group in the domestic poultry population. There are also vast numbers of domestic ducks in Egypt in the household sector, a recognized source of infection and transmission of the HPAI H5N1 virus to poultry and humans [3–7]. Previous research suggests that water fowls (migratory and domestic) play a crucial role in the perpetuation and dissemination of avian influenza (AI) viruses globally [3–8]. It is therefore crucial to mitigate the risk of HPAI H5N1 infection and to reduce the circulation of HPAI in domestic ducks to control the spread of HPAI H5N1 [9]. Vaccination can decrease disease prevalence and reduce viral shedding among infected poultry, thereby decreasing the rate of environmental contamination, especially where enforcement of biosecurity is impractical [9]. Although AI vaccination has been widely implemented as a disease control tool in Egypt, very little or no post-vaccination monitoring has taken place in the country [10–12]. In addition, there is insufficient information on the effectiveness of HPAI H5N1 vaccination in domestic duck species to guide disease control programs. Since the licensing of a new live recombinant vector vaccine (rHVT-H5) based on a turkey herpes virus (HVT) expressing the H5 gene from a clade 2.2 HPAI H5N1 virus in Egypt late in 2012, the vaccine has gained acceptance [12]. Furthermore, because so far no AI vaccine has been developed specifically for water fowl, all the available studies for this vaccine have been on specific pathogen-free (SPF) chickens [13–15], commercial broilers [12,16], and commercial layers [12] only. Because the ducks play a central role in the epidemiology of HPAI H5N1 in Egypt, the present study was conducted to fill the knowledge gap about the rHVT-H5 vaccine and to assess the effectiveness of HPAI H5N1 vaccine in domestic water fowl. The study was specifically designed to monitor the post-vaccination serological response to a single dose of the rHVT-H5 vaccine, compared to a dose of an inactivated H5N1 reverse genetic vaccine based on clade 2.2.1 and 2.2.1.1 Egyptian seed strain (MEFLUVAC), in protecting domestic Mulard ducks from challenges with HPAI H5N1 clade 2.2.1 viruses from Egypt.

## Results

### Maternally-derived antibody (MDA) monitoring

**Fig 1:**
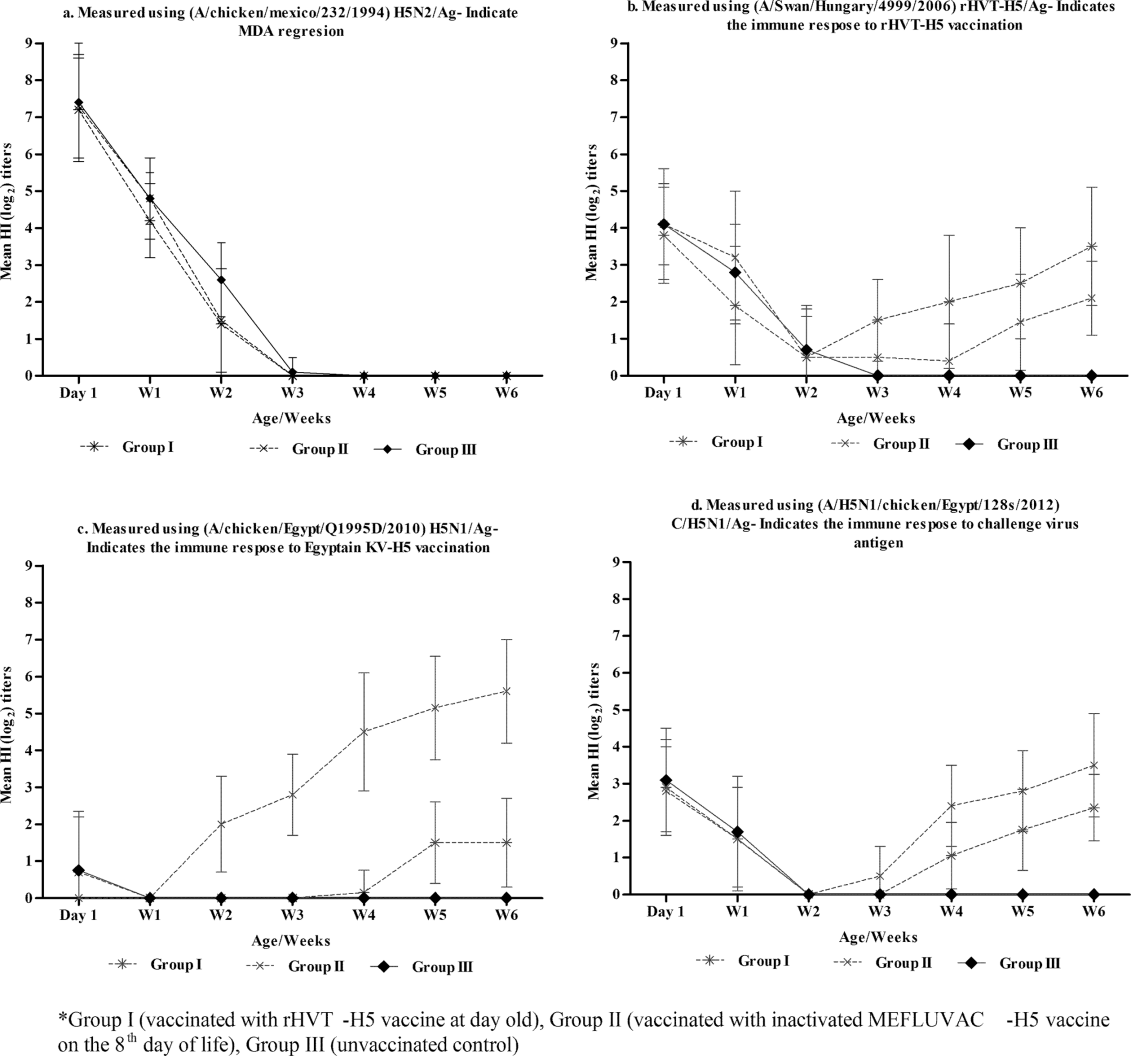
Weekly mean HI titres (log2 ± SD) showing the results of MDA regression profiles and post vaccination immune responses in different experimental groups*. Weekly Mean HI titers (log 2) measured using (**1a**) (A/Chicken/Mexico/232/1994) H5N2/Ag indicating MDA regression; (**1b**) (A/Swan/Hungary/4999/2006)rHVT-H5/Ag indicating immune response to rHVT-H5 vaccination; (**1c**) (A/Chicken/Egypt/Q1995D/2010)H5N1/Ag indicating immune response to KVT-H5 vaccination; (**1d**) (A/H5N1/Chicken/Egypt/128s/2012)C/H5N1/Ag indicating immune response to challenge virus antigen. *Group I (1b) (vaccinated with rHVT-H5 vaccine at 1 day old), *Group II (1c) (vaccinated with inactivated MEFLUVAC-H5 vaccine at 8 days old)*, *Group III (1d) (unvaccinated control)*.

**Fig 2:**
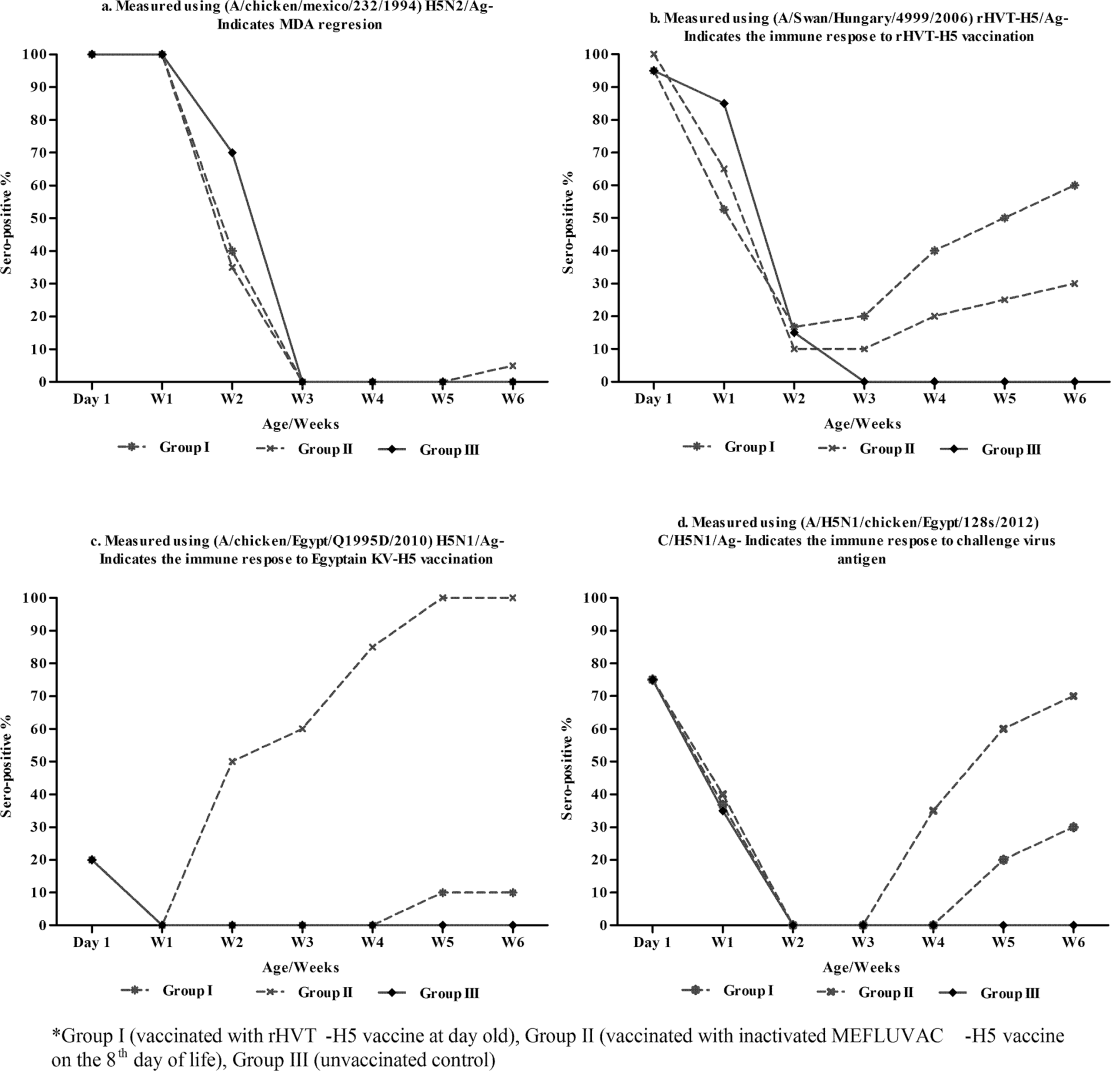
Weekly sero-conversion (%) showing the results of MDA regression profiles and post vaccination immune responses in different experimental groups*. Weekly Mean Sero-conversion (%) measured using (**2a**) (A/Chicken/Mexico/232/1994) H5N2/Ag indicating MDA regression; (**2b**) (A/Swan/Hungary/4999/2006)rHVT-H5/Ag indicating immune response to rHVT-H5 vaccination; (**2c**) (A/Chicken/Egypt/Q1995D/2010)H5N1/Ag indicating immune response to KVT-H5 vaccination; (**2d**) (A/H5N1/Chicken/Egypt/128s/2012)C/H5N1/Ag indicating immune response to challenge virus antigen. *Group I (vaccinated with rHVT-H5 vaccine at 1 day old), Group II (vaccinated with inactivated MEFLUVAC-H5 vaccine at 8 days old), Group III (unvaccinated control).

We obtained 225 commercial Mulard day-old-ducks from breeder flock that has been vaccinated four times using the H5N2 vaccine. During the first week, all the tested ducklings from the two experimental groups (n = 15/group) and the controls (n = 15) were 100% seropositive. At one day old, the mean HI titres of the MDAs ranged from 7.3 to 7.4 log_2,_ using H5N2/Ag (Table 1, Fig 1A). However, other antigens did not perform as well as the H5N2/Ag (Fig 1B–1D, S1–S3 Tables). The MDA titres decreased constantly until the third week in the two vaccinated groups (the rHVTH5-vaccinated and MEFLUVAC-H5-vaccinated groups) and the control. (Table 1, Fig 1A). However, while the MDA decreased in the other groups, it continued to exist until the fourth week in the unvaccinated controls (Table 1). Only during the second week was the MDA mean titre for Group III significantly higher (p<0.05) than the MDA titre for the vaccinated groups (I and II) (Table 1). In this study, the time to reach the half-life of MDAs was <7 days for vaccinated Group I (5.9 days) and Group II (6.1days), and it was 9.3 days for the unvaccinated (Group III) birds (Fig 2A–2D).

**Table 1:** Weekly HI mean titres (log2 ± SD) using (A/chicken/Mexico/232/1994) H5N2/Ag that indicate the MDAs’ regression profile.

Age (Weeks)	Group*		
	I	II	III
0 (day 1)	7.3 ±1.4^a^	7.4±1.4^b^	7.4±1.6^b^
1	4.2±1^a^	4.8±1.2^b^	4.8±0.7^b^
2	1.4±1.5^a^	1.5±1.4^a^	2.6±1^b^
3	0±^0a^	0±0^a^	0.1±0.4^a^
4	0±0^a^	0±0^a^	0±0^a^
5	0±0^a^	0±0^a^	0±0^a^
6	0±0^a^	0±0^a^	0±0^a^

Different superscripts in a row denote the presence of statistically significant (p ≤0.05) differences.

*Group I (vaccinated with rHVT-H5 vaccine at 1 day old), Group II (vaccinated with inactivated KV-H5 vaccine at 8 days old), Group III (unvaccinated control).

### Post-vaccination antibody response to the rHVT-H5 vaccine

The three experimental groups were highly sero-positive (90–95%) during the first week, but the mean HI titres of the MDA were significantly lower than where the H5N2/Ag was used (Fig 2A, Table 2). The detected HI mean titres of the MDA ranged from 3.8 to 7.4 log_2_ (Table 2, Fig 2A). The post-vaccination antibody response against the rHVT-H5 in Group I started from the second week, with a sero-conversion rate of 16.7% (Table 2, Fig 2B). The post-vaccination immune response continued in its development on a weekly basis until the HI mean titres reached 3.5log_2_ and 70% sero-conversion by the sixth week (Table 2 and Fig 2A–2D).

**Table 2:** HI titres* (mean ±SD) and sero-positivity rates for the selected ducks subject to the four challenge experiments**.

Age	Parameters	Group I (n = 10)		Group II (n = 10)		Group III (n = 10)	
		rHVT Ag	H5N1/2012	H5N1/2010	H5N1/2012	H5N2	H5N1/2012
1 day	HI log_2_ titres	3.8±1.3^a^	2.9±1.3^a^	0.8±1.6^b^	2.8±1.2^a^	7.4±1.6^c^	3.1±1.4^a^
	Sero-conversion (%)	95	75	20	75	100	75
7 days	HI log_2_ titres	1.9±1.6^a^	1.5±1.4^a^	0±0^b^	1.5±1.4^a^	4.8±0.7^c^	1.7±1.5^a^
	Sero-conversion (%)	52	36.8	0	40	100	35
14 days	HI log_2_ titres	0.5±1.1^a^	0±0^a^	2.8±1.1^b^	0±0^a^	2.6±1^b^	0±0^a^
	Sero-conversion (%)	16.7	0	50	0	70	0
21 days	HI log_2_ titres	1.5±1.6^a^	0±0^b^	2.8±0.5^c^	1.7±0.4^a^	0.1±0.4^b^	0±0^b^
	Sero-conversion (%)	20	0	60	0	0	0
28 days	HI log_2_ titres	2±1.4 ^a^	1.3±1.4 ^b^	4.5±1.1 ^c^	1.7±1.4 ^b^	0±0 ^d^	0±0 ^d^
	Sero-conversion (%)	40	20	85	35	0	0
35 days	HI log_2_ titres	2.5±0.5 ^a^	1.8±1.3 ^b^	5.2±0.9 ^c^	2.8±0.8 ^a^	0±0 ^d^	0±0 ^d^
	Sero-conversion (%)	60	20	100	60	0	0
42 days	HI log_2_ titres	3.5±0.5^a^	2.4±0.5^b^	5.6±0.7^c^	2.8±0.5^b^	0±0^e^	0±0^e^
	Sero-conversion (%)	70	30	100	70	0	0

Different superscripts in a row denote the presence of statistically significant (p ≤0.05) differences

*HI titres were measured using homologous vaccinal antigens: rHVT-H5/Ag (A/Swan/Hungary/4999/2006) was used for Groups I; V/H5N1/Ag (A/chicken/Egypt/Q1995D/2010) was used for Group II, and H5N2/Ag (A/chicken/Mexico/232/1994) was used for Group III.

**Group 1 (vaccinated with rHVT-H5 vaccine at 1 day old), Group II (vaccinated with inactivated KV-H5 vaccine at 8 days old), Group III (unvaccinated control). All birds were challenged with HPAI H5N1 virus (A/Chicken/Egypt/128S/2012 (H5N1)) (GenBank Accession No. JQ858485) via the oculo-nasal route with 100 μl (50_ul in eye, 50_ul in nose) of inoculum containing 10^6^ EID_50_ per duck.

### Post-vaccination antibody response to MEFLUVAC-H5 vaccine

Using the V/H5N1/Ag as the homologous antigen to the MEFLUVAC–H5 vaccine, the MDA monitoring indicated that in the three experimental groups, significantly lower (undetectable level of antibodies (range 0.0 to 1.5 log_2_) existed in the first week post vaccination until the third week. The antibody titres gradually developed thereafter with higher titres obtained in group II (Table 2, Fig 2C and S2 Table). The post-vaccination antibody response against the MEFLUVAC-H5 in Group II started from the second week with a sero-conversion rate of 50%. The post-vaccination immune response continued to develop on a weekly basis until the HI mean titres reached 5.6log_2_ and 100% sero-conversion by the sixth week (Table 2, Fig 2C).

### Post-vaccination antibody response against the HPAI H5N1 challenge virus antigen (C/H5N1/Ag)

All the experimental groups showed (≥75%) sero-positivity at Day 1. The MDA mean HI titres (0.8–3.1 log_2_) were significantly lower for H5N1/Ag than where the H5N2/Ag was used (Table 2, S1–S3 Tables). MDA waned significantly by the end of the second week in all three experimental groups (Figs 1 and 2). The post-vaccination antibody response against the C/H5N1/Ag in Group II started in the third week, while in Group I, it started in the fourth week (S3 Table, Fig 2D). The post-vaccination mean antibody titre detected in Group I and II were (0±0, 1.1±1.3, 1.8±1.3 and 2.4±1.1 log_2_) and (1.7±1.3, 2.4±1.4, 2.8±1.1 and 3.5±1.1log_2_) at third, fourth, fifth and sixth weeks respectively (S3 Table). The sero-conversion rates detected in Group I and II at the third, fourth, fifth and sixth weeks gradually peaked (Table 2). In general, the mean HI antibody titres and sero-conversion rates were significantly higher in Group II than in Group I at the third, fourth, fifth and sixth weeks (Table 2 and Fig 2).

### Challenge study: Clinical signs and protection

#### Control birds

The control positive challenged group displayed 100% morbidity during the four different challenge experiments at 21, 28, 35 and 42 days (Table 3). All the challenged control positive groups displayed depression, pink eye, respiratory distress and nervous manifestations before death. There was no clear difference between the means for the onset of clinical signs between the four different control positive groups, although the trend displayed by age (21, 28, 35 and 42 days) differed, as the mean duration of the onset of clinical signs increased (1.1, 1.2, 1.3 and 1.5 days) respectively (Table 3). The mean duration of the clinical signs (the patent period) also displayed the same age trend in the four challenge experiments were 7, 6, 8.7 and 11.3 days respectively (Table 3). The control positive challenged groups displayed 100% mortality and a 100% case fatality rate (CFR). However, the mean death time (MDT) was 8.2, 8, 9.8 and 12.8 days respectively during the four challenge experiments, suggesting the possibility of age susceptibility (Table 3).

**Table 3:** Morbidity and mortality parameters measured in animals subjected to four separate challenge experiments (Exp.1, 2, 3 and 4)*.

Parameters	Group I *(n = 10)*				Group II *(n = 10)*				Group III *(n = 10)*			
	Exp. 1	Exp. 2	Exp.3	Exp. 4	Exp. 1	Exp. 2	Exp.3	Exp. 4	Exp. 1	Exp. 2	Exp.3	Exp. 4
**Morbidity (%)**	100	100	100	100	100	100	100	100	100	100	100	100
Mean duration for clinical onset (days) (range)	2.7(1–5)	4.2(1–6)	3 (1–5)	3.4(2–4)	3.1(2–4)	3.4(2–5)	3(2–5)	2.9(2–4)	1.1(1–3)	1.2(1–2)	1.3(1–2)	1.5(1–2)
Overall mean patent period (days) (range)	10.1(7–12)	6(6–12)	10.1(8–12)	10.1(9–12)	7(5–8)	9.2(4–12)	9.7(6–13)	9.2(7–12)	7	6	8.76–13)	11.3(9–13)
Mean patent period (days) for birds that died (range)	7	7.5(7–8)	11(9–13)	12	7(6–8)	5.3(4–6)	8	12	7	6	8.7(5–13)	11.3(9–13)
Mean patent period (in days) for birds that survived (range)	10.9(10–12)	5.6(4–8)	9.9(9–11)	9.9(9–11)	7(5–8)	10.9(9–12)	10.1(8–12)	8.9(7–12)	NA	NA	NA	NA
**Mortality (%)**	20	20	10	10	50	30	20	10	100	100	100	100
Total no. sick birds but recovered (%)	80	80	90	90	50	70	80	90	0	0	0	0
Case fatality rate (%)	20	20	10	10	50	30	20	10	100	100	100	100
Mean death time (MDT)	8	12	14	14	7.8	9.3	10	14	8.2	8	9.8	12.8
**Protection (%)**	80	80	90	90	50	70	80	90	0	0	0	0

*Experiments 1, 2, 3 and 4 were carried out on birds of 21, 28 and 35 and 42 days of age. In both cases, Group 1 (vaccinated with rHVT-H5 vaccine at 1 day old), Group II (vaccinated with inactivated KV-H5 vaccine at 8 days old), Group III (unvaccinated control). All birds were challenged with HPAI H5N1 virus (A/Chicken/Egypt/128S/2012 (H5N1)) (GeneBank Accession No. JQ858485) via the oculo-nasal route with 100 μl (50_ul in eye, 50_ul in nose) of inoculum containing 10^6^ EID_50_ per duck.

#### Group I (rHVT-H5 vaccinated group)

Group I displayed 100% morbidity during the four different challenge experiments. All the challenged birds displayed depression, pink eye and mild respiratory distress, and nervous manifestations appeared only in these birds that died. There was no difference in the mean duration for the onset of clinical signs (2.7, 4.2, 3.0 and 3.4 days) between the four experiments (21, 28, 35 and 42 days), although it was longer than for the control positive groups (Table 3). The mean duration of clinical signs (patent period) also was 10.1, 6, 10.1 and 10.1 days, during the four challenge experiments respectively. Group I challenge birds showed 20%, 20%, 10%, and 10% *mortality* and *CFR* was 20%, 20%, 10% and 10% respectively in the four different experiments. However, the mean death time (MDT) was 8, 12, 14 and 14 days respectively during the fourth challenge experiment. In terms of the *protection percentage* (against mortality), the rHVT-H5-vaccinated birds displayed the highest protection levels at 80%, 80%, 90% and 90% respectively during the four challenge experiments (Table 3).

#### Group II (MEFLUVAC-H5 vaccinated group)

Group II challenged birds displayed 100% *morbidity* in the four different challenge experiments. All the challenged birds displayed depression, pink eye, and mild respiratory, but nervous manifestation appeared only in the birds that died. There was no difference in the means for the onset of clinical signs (3.1, 3.4, 3 and 2.9 days) between the four experiments (21, 28, 35 and 42 days of age), although it was longer than in the control positive groups (Table 3). The mean duration of clinical signs (patent period) was also 7, 9.2, 9.7 and 9.2 days respectively during the four challenge experiments. Group II challenge birds displayed 50%, 30%, 20% and 10% *mortality*, and *CFR* was 50%, 30%, 20% and 10% respectively in the four different experiments. However, the mean death time (MDT) was 7.8, 9.3, 10 and 14 days respectively in the fourth challenge experiment (Table 3). The *percentage of* protection against mortality was highest for the MEFLUVAC-H5-vaccinated birds, at 50%, 70%, 80% and 90% respectively during the four challenge experiments (Table 3). In general, Group I performed the best during the first two challenge experiments, and there was no difference between the two vaccinated groups in the third and fourth challenge experiments.

### Viral shedding

#### Rate of viral shedding

The control positive groups (Group III) displayed a 100% shedding rate in the four different challenge experiments. All the shedder birds in the control positive groups died.

In the case of Group I, the shedding rates were 80%, 40%, 40% and 20% respectively in the four challenge experiments (Table 4). The number of shedder birds that died was 2, 2, 2 and 1 respectively in the four challenge experiments. The total number of sick birds that did not shed the virus was 2, 6, 6 and 8 birds respectively.

**Table 4:** Virological parameters measured in animals subjected to four separate challenge experiments (Exp.1, 2, 3 and 4)*.

Parameters	Group I *(n = 10)*				Group II *(n = 10)*				Group III *(n = 10)*			
	Exp. 1	Exp. 2	Exp.3	Exp.4	Exp. 1	Exp. 2	Exp.3	Exp.4	Exp. 1	Exp. 2	Exp.3	Exp.4
Total no. shedder birds (%)	8 (80%)^a^	4 (40%)^b^	4 (40%) ^b^	2 (20%)^c^	5 (50%) ^d^	8 (80%) ^a^	8 (80%) ^a^	2 (20%) ^c^	10 (100%) ^e^	10 (100%) ^e^	10 (100%) ^e^	10 (100%) ^e^
Shedder birds that died	2	2	2	1	5	3	2	1	10	10	10	10
Shedder birds that recovered	6	2	2	1	0	5	6	1	0	0	0	0
Total no. sick birds but did not shed virus	2	6	6	8	5	2	2	8	0	0	0	0
Mean virus load/ shedder birds												
Third dpc	4.1±1.2	2.7±0.4	3.6±0.3	3.1±0.1	3.3±0.2	3.6±1.2	3.9±1.3	3	3.9±1	3.5±0.3	4.4±0.4	3±0.5
Sixth dpc	3.2±0.4	3.2±0.1	4.1±0.7	2.0±0.1	3.5±1.1	3.2±0.3	3.1±1	2.4±0.6	3.6±0.6	3.6±0.5	4.1±0.8	3.5±0.3
Ninth dpc	0	0	4.2	0	3.3±0.3	4.5±0.3	4.5±0.2	3.7	**NA	NA	3.8±1.2	4±0.5
Fourteenth dpc	0	0	2.5	0	0	0	0	3.9	NA	NA	5±0.7	4.8±0.8
Mean virus load/shedder birds that died	3.9 ±0.5	3.5	3.4±1.2	3.4	3.6±0.5	4.5±0.2	4.5±0.2	3.9	4.7±0.7	5.1±0.5	5.4±0.6	4.7±0.7

Different superscripts in a row denote the presence of statistically significant (p ≤0.05) differences

*Experiment 1, 2, 3 and 4 were carried out on birds of 21, 28, 35 and 42 days of age. In both cases, Group 1 (vaccinated with rHVT-H5 vaccine at 1 day old), Group II (vaccinated with inactivated KV-H5 vaccine at 8 days old), Group III (unvaccinated control). All birds were challenged with HPAI H5N1 virus (A/Chicken/Egypt/128S/2012 (H5N1)) (GeneBank Accession No. JQ858485) via the oculo-nasal route with 100 ul (50_ul in eye, 50_ul in nose) of inoculum containing 10^6^ EID_50_ per duck.

** NA = not applicable as all birds died before 10 dpc.

In case of Group II, the shedding rates were 50%, 80%, 80% and 20% respectively during the four challenge experiments. The number of shedder birds that died was 5, 3, 2 and 1 respectively during the four challenge experiments. The total number of sick birds that did not shed the virus was 5, 2, 2 and 8 birds respectively (Table 4).

In general, the highest shedding rate (80%) was recorded in Group I in the first challenge experiment. In Group II, it was recorded in the second and third challenge experiments. The rate of shedding decreased by age in both vaccinated groups. In the fourth challenge experiment, both vaccinated groups displayed the same trend (Table 4).

#### Amount of challenge viral shedding (log_10_ PCR copies)

In Group III, the mean viral shedding in the control positive groups ranged from 3.6 to 5 log_10_/ml in the surviving birds. The mean viral shedding in dead birds was 4.7±0.7, 5.1±0.5, 5.4±0.6 and 4.7±0.7 log_10_/ml respectively in the four different experiments. The control positive birds in the third and fourth challenge experiment continued shedding until the 14th dpc. The highest shedding rates (5±0.7 and 4.8±0.8) among the four challenge control positive groups were detected in the third and fourth challenge experiments in late shedding birds respectively (Table 4).

In Group I, the mean viral shedding in Group I-challenged birds during the four challenge experiments ranged from 2 to 4.1 log_10_/ml in the surviving birds. The mean viral shedding in dead birds was 3.9±0.5, 3±0, 3.4±1.2 and 3.4 log_10_/ml in the four different experiments respectively. Viral shedding stopped from the sixth dpc in the first, second and fourth challenge experiments, but it continues until the 14^th^dpc in the third challenge experiment. The level of viral shedding decreased as the age of the challenged birds increased. The mean viral shedding in Group I challenged birds in the four challenge experiments was lower than in the case of the control positive groups by 1 to 2 log_10_ (Table 4).

In Group II, the mean viral shedding in Group II-challenged birds during the four challenge experiments ranged from 2.4 to 4.5 log_10_/ml in the surviving birds. The mean viral shedding in dead birds was 3.6±0.5, 4.5±0.2, 4.5±0.2 and 3.9 log_10_/ml in the four different experiments respectively. Viral shedding stopped from the 9^th^dpc in the first, second and third challenge experiments, but continued until the 14^th^dpc in the fourth challenge experiment. The mean viral shedding in Group II challenged birds in the four challenge experiments was lower than in case of the control positive groups by 1 log_10_ (Table 4).

In Group III, the mean viral shedding in Group III-challenged birds during the four challenge experiments ranged from 4.7to 5.4 log_10_/ml in the surviving birds. The mean viral shedding in dead birds was 4.7±0.7, 5.1±0.5, 5.4±0.6 and 4.7±0.7 log_10_/ml in the four different experiments respectively. Viral shedding stopped became undetectable from the 6^th^dpc in the first and second challenge experiments, but continued until the 14^th^dpc in the third and fourth challenge experiments (Table 4).

## Discussion

In the winter of 2014–2015, Egypt recorded an epizootic of HPAI H5N1 virus, because of the emergence of a new but dominant H5N1 virus cluster (clade 2.2.1.2) [17]. Prior to this event, Egypt’s poultry population has continued to be infected with the H5N1 virus with regular reports of human cases. To date, the following control measures have been implemented to mitigate the extent and impact of avian influenza H5N1: biosecurity, a ban on poultry movement, market rest days, stamping out, and vaccination [18]. Whereas the first four measures are perceived as the classical approach to the disease and have gained prominence worldwide, vaccination against avian influenza H5N1 is still hotly debated and its implementation is considered on a country-by-country basis only. Because Egypt has been declared an area where the virus is endemic to the poultry population and multiple human infections have been reported, the country has adopted vaccination of poultry against avian influenza to increase resistance to infection, to protect the poultry population from clinical disease, and to reduce shedding of the virus in infected birds.

Many vaccines against the H5N1 virus have been used and studied [19–23], however, no single study has considered the efficacy of influenza H5N1 vaccine in Mulard ducks. This species of domestic duck is a reproductive sterile hybrid between Perkin ducks (*Anasplatyrhynchos domestica*) and muscovy ducks (*Cairina moschata*), and this hybrid is susceptible to infection and can effectively transmit the virus [24].

Previous work by Cagle et al. [20], confirmed that both Perkin and Muscovy ducks have received efficient vaccination for protection against the spread of the disease, but suggested that they respond differently to the HPAI H5N1 commercial inactivated vaccine. It is therefore difficult to generalize for duck species, based on these previous evaluations. In addition, the virus may still be shed even in clinically healthy vaccinated populations [20], particularly in the summer months [25, 26]. Furthermore, although previous studies have confirmed the effectiveness of inactivated H5 vaccines in protecting ducks against disease [27–29], continuous shedding of the virus occurs in clinically healthy vaccinated duck populations [30], and low scale transmission to other poultry can still occur. Current H5 inactivated vaccines and vaccination practices are thus insufficient to control H5N1 HPAI virus infections in domestic waterfowl. New vaccination strategies are thus needed to improve the level of protection for ducks.

In this study, we have demonstrated the protective efficacy of a commercial live recombinant vector vaccine (rHVT-H5) given on its own, and have compared it with using an inactivated H5N1 vaccine (MEFLUVAC), or no vaccination, in domestic Mulard ducks (a cross-breed between Muscovy and mallard breeds). We used a virulent challenge virus that belongs to major HA clade 2.2.1 circulating in Egypt and that is known to have previously caused severe clinical signs and 100% mortality in non-vaccinated ducks.

The ducklings used in this study had maternally derived antibodies (MDA) from previous multiple vaccination of the breeder ducks. However, we have determined that such MDA waned significantly on or before 21 days of age. Decision-making on vaccination against HPAI H5N1 in ducks should consider such degrading immunity before vaccination, because interference between MDA and the protection provided by avian influenza inactivated vaccines has been reported in poultry [12, 13, 22, 31]. In this case, the MDA half-life was less than 7 days in the case of the rHVT-H5 and inactivated H5N1 vaccinated groups (5.9 and 6.1 days respectively), while it was 9 days for non-vaccinated birds. Our work suggests that vaccination at 7 days of age could help minimize the interference of maternal antibodies, and would enable the ducks to acquire early immunity. Despite this immunity and good protection against the disease, viral shedding still persisted in these species [29].

In this study, the ducks vaccinated with the rHVT-H5 and inactivated MEFLUVAC vaccines rapidly developed antibodies and were well protected against the challenge with the Egyptian HPAI H5N1 virus. However, most ducks shed the virus for at least six days in the case of the rHVT-H5-vaccinated groups, and for up to nine days in the case of the groups inoculated with the inactivated vaccine. Although virus titres were generally lower than the titres observed in the challenge controls for both groups of ducks, neither the rHVT-H5 vaccine nor the inactivated vaccine were capable of protecting challenged ducklings from displaying clinical signs. However, the vaccinated duck groups had a higher mean death time than the challenge controls, and this difference in survival was significant (P>0.05). Ducks vaccinated with the inactivated vaccine challenged with the Egyptian virus shed the virus for a longer period than those vaccinated with the rHVT-H5 vaccine.

The rHVT-H5 vaccine induced a weak antibody response to its homologous antigen and undetectable level for other viruses tested, indicating that it is most likely to replicate in Mulard ducks [16]. In fact, the virus was detected in tissues from rHVT-H5-vaccinated ducks when they were examined at 14 and 21 days after vaccination, both in feather follicles and in the spleen [16, 32].

In this study, we have confirmed that both vaccines were effective and conferred protection. In addition, they induced both cellular and humoral immune responses, including cell-mediated immunity [15]. Several studies have been conducted to evaluate vaccine efficacy in ducks against a HPAI virus lethal challenge [27–29]. Vaccine protection from infection and the level of viral shedding varied depending on single or double-dose vaccination [30], and the challenge virus strain [29].

The majority of the published vaccine studies on ducks were done either on Perkin ducks (*Anasplatyrhynchos domesticus*) [5,27,33], or mallard ducks (*A*. *platyrhynchos*) [34,35] and less research has been done on Muscovy ducks (*Cairinamoschata*) [33,36], even though Muscovy ducks are economically significant, as they are not produced only in Asia, but also represent 90% of the ducks hatched in France, the primary producer of ducks in Europe [36]. Vaccine efficacy studies in ducks conducted by Steensels et al. [33] using fowlpox-vectored AI vaccination (TROVAC AIV H5, rFP-AIV-H5) revealed oropharyngeal virus shedding in Muscovy ducks as late as 19 days after infection (dpi), while no shedding was detectable in Perkin ducks at any point after infection with the same HPAI H5N1virus. However, no research has been done comparing the responses of Perkin and Muscovy ducks to vaccination in the same study and under the same conditions.

In conclusion, the rHVT-H5 vaccine used alone and with inactivated H5 vaccine could confer the required level of protection in Mulard ducks when they are challenged with the HPAI H5N1 clade 2.2.1 viruses. The rHVT-H5 vaccine appears to offer better protection than the inactivated vaccine, although it produced a higher level of viral shedding until Day 6 after the challenge. Vaccinating ducklings with the rHVT-H5 vaccine should reduce the transmission of HPAI H5N1 among domestic poultry.

## Materials and Methods

**Fig 3:**
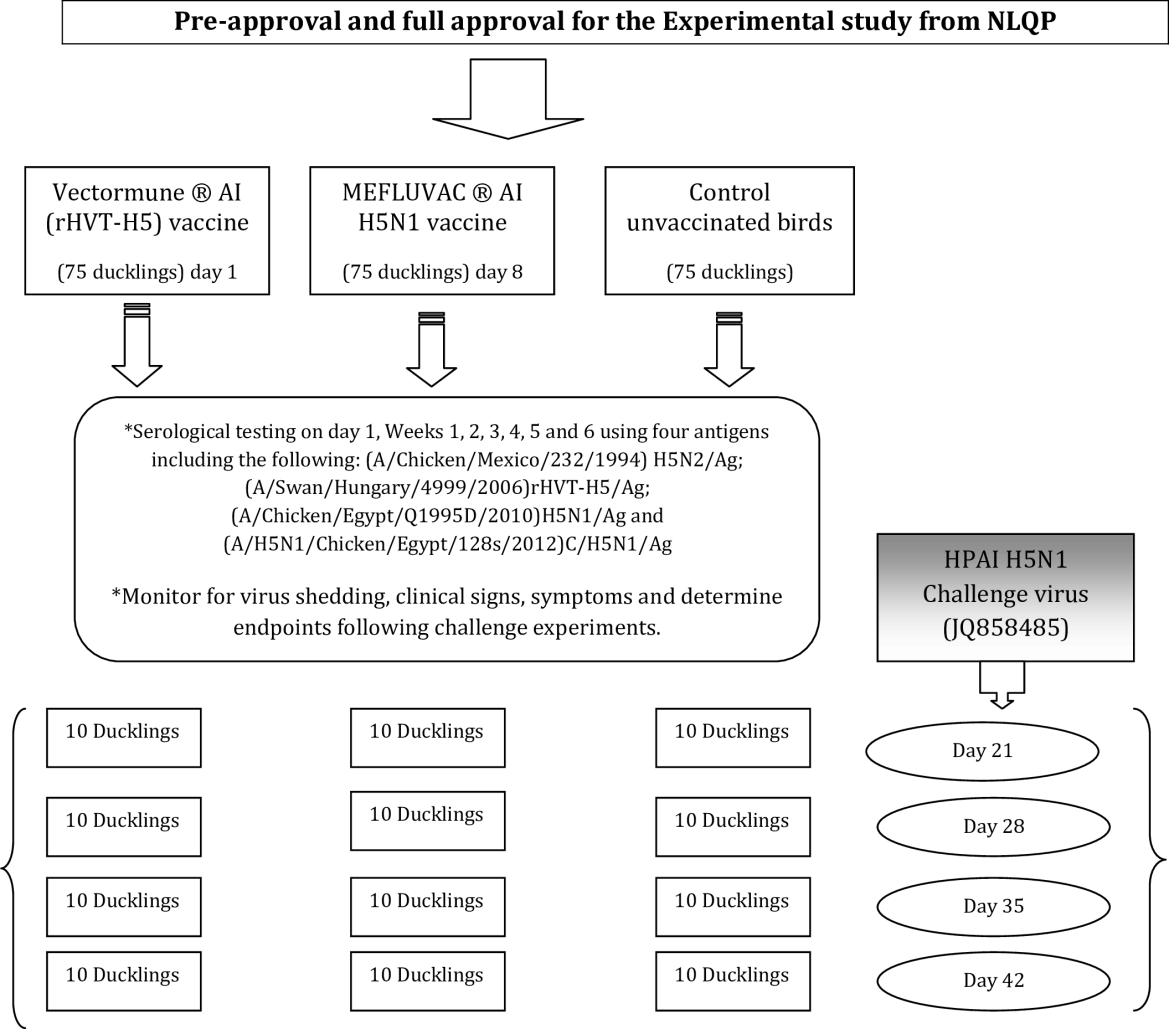
Model of experimental design.

All studies were carried out according to the Reference Laboratory for Veterinary Quality Control on Poultry Production (NLQP) guidelines for research ethics in animals. The project ethics approval number was LA140801/Ex04. Model of experimental design are described in Fig 3.

### Vaccines and Challenge virus

#### Vectormune® AI (rHVT-H5)

It is a cryopreserved cell-associated recombinant turkey herpes virus constructed with the FC-126 strain of HVT as the vector, and the HA gene from the H5N1 clade 2.2 HPAI (A/Swan/ Hungary/ 4999/2006) strain as an insert, and manufactured by CEVA-Santé animalé (Libourne, France). The details of the vaccine have been described in previous studies [16, 22]. It is injected subcutaneously in day-old birds, usually in the lower third of the neck at a dose rate of 12,450 plaque forming units (pfu)/0.3 ml/duckling (the equivalent of 1.5 chicken doses). The Serial number of the vaccine was 395–042 with expiration date set at 09 October 2016.

#### Egyptian inactivated bivalent H5N1 vaccine (MEFLUVAC)

It is an oil emulsion inactivated vaccine that contains two reassortant H5N1 strains (A/chicken/Egypt/Q1995D/2010; GeneBank Accession No. CY099579 and A/duck/Egypt/M2583D/2010; GeneBank Accession No. CY099580). The inactivated bivalent vaccine was manufactured by the Middle East Vaccine Producing Company (MEVAC) (Al Sharkia-Egypt). It is administered at a dose of 0.5 ml per bird I/M as per manufacturer’s recommendation for chicken. The Batch number of the vaccine was 1402250101 with expiration date set at 25 February 2016.

#### HPAI H5N1 virus (A/chicken/Egypt/128s/2012(H5N1) (GeneBank Accession No. JQ858485)

The HPAI H5N1 virus (A/chicken/Egypt/128s/2012(H5N1) (HA clade 2.2.1) was obtained from the repository of the Reference Laboratory for Veterinary Quality Control on Poultry Production (NLQP), Animal Health Research Institute, Giza, Egypt. The virus was inoculated into the allantoic cavity of 9-day-old embryonating chicken eggs and grown for 30 hours at 37°C. The allantoic fluid was harvested and frozen at −70°C until further use.

### Study protocol

A total of 225 commercial day-old Mulard ducks (DODs) that carried maternally derived antibodies (MDAs) against H5N2 vaccine were obtained from a commercial duck breeder (Al-Wafaa Company, Egypt). The duck breeder flocks were vaccinated four times against avian influenza H5, at Weeks 2, 8, 19 and 40, using different H5N2 inactivated vaccines. The DODs were kept at biosecurity level 3 (BSL-3) poultry isolators at the Reference Laboratory for the Quality Control of Poultry Production (NLQP) animal facility. The ducks for this experiment were allocated into three groups (I, II and III) of 75 ducklings each. At one day old, 15 DODs from each group were humanely bled intra-cardially to monitor for MDA titres. The Group I ducklings (n = 75) were vaccinated with rHVT-H5 vaccine at one day old; Group II ducklings (n = 75) received MEFLUVAC-H5 vaccine when they were eight days old, and Group III (n = 75) served as unvaccinated controls. All the experimental DODs were monitored daily for 56 days.

### Serological monitoring

Fifteen (15) randomly selected birds from each group were bled at Day 1, and at Weeks 1 2, 3, 4, 5 and 6. Sera were individually identified by group and date, and stored at -20°C until tested. Duck antisera were treated using 10% chicken RBCs before HI testing was conducted to avoid any non-specific reactions [37], The haemagglutination inhibition (HI) test was conducted according to standard procedures and as previously described by the World Organisation for Animal Health [22,37]. Briefly, an HI test was conducted using four different H5 antigens: i) the H5N2/Ag (A/chicken/Mexico/232/1994 (H5N2) (Genebank Accession No. AY497096) to determine MDA titres; ii) the rHVT-H5/Ag, homologous vaccinal antigen (A/Swan/Hungary/4999/2006) to determine post-vaccination antibody immune response against the rHVT-H5 vaccine; iii) the V/H5N1/Ag,homologous Egyptian H5N1 antigen (A/chicken/Egypt/Q1995D/2010) (GeneBank Accession No. CY099579) to determine any post-vaccination immune response against the MEFLUVAC-H5 vaccine, and iv) the C/H5N1/Ag challenge virus antigen (A/H5N1/chicken/Egypt/128s/2012) (GeneBank Accession No. AFI 44355) to evaluate the post-vaccination immune response against the challenge virus. The HI mean titres were expressed as reciprocal log_2,_ and an HI titre in a dilution of >2^3^ was used to test specifically for the presence AI H5 antibody titres. The sero-conversion (sero-positivity) rate was estimated as the proportion of birds with positive HI titres (≥2^3^) and was calculated using the following formula:

$$\frac{n}{N}\times \frac{100}{1}$$

where n = number positive with HA titres of ≥2^3^; N = total randomly selected and tested number in the group.

### Detection of rHVT-H5 in vaccinated birds

The rHVT-H5 virus replication was assessed in both the spleen and feather follicles of rHVt-H5 vaccinated and control (non-vaccinated) birds at the age of 14 and 21 days. Specifically, the Marek’s disease virus serotype 3 (HVT) US3 gene was targeted using the PCR test according to Handberg *et al*.[38].

### The challenge experiment

Considering the lack of standardized information on the challenge and protection time for AI vaccine evaluation in ducks, and the differential responses in different species of ducks [20], four different sets of challenge experiments were conducted at Days 21, 28, 35 and 42. In each challenge trial, 10 DODs, tagged and identified individually, were randomly selected and allocated from each group (Groups I, II and III). The birds were transferred to separate BLS-3 poultry isolators, and a challenge experiment was conducted according to the NLQP guidelines for research ethics in animals. All experimental infections were performed via the oculo-nasal route, with 100 μl of the challenge inoculum, containing 10^6^ EID_50_/duckling (with 50μl administered into the eye and 50μl into the nasal cavity).

### Animal care and welfare during the study

All ducks were placed under a 24-hour monitoring program by the laboratory animal facility team for the duration of the study and no incidence of animal death or unexpected occurrence was recorded in the course of the experiment.

However, in the course of the challenge experiment with highly pathogenic avian influenza virus, it is expected that some ducks may die, and humane endpoint protocol were submitted and approved by the Institution Ethics Committee for euthanasia of such animal. Situation that will trigger the use of humane endpoint include the display of severe clinical signs that cause suffering (Score of ≥ 6) [39], pain or incompatible with animal welfare including prostration and nervous manifestation that affects normal movement and food intake and respiration inside the isolator for 24–48 hours.

Euthanasia of individual experimental bird was performed by the intra-vascular inoculation of sodium pentobarbital (100 mg/kg) with or without CO_2_ gas. This medication was provided in a small ready-to-use vials (0.324 grams/ml) for use in individual experiments. A dose of 0.1–0.2 ml was administered intravascularly to each 4-to-6 week old bird to quickly cause unconsciousness and death for an experimental duck that was severally sick as described above. At the end of the experiment, all survivor ducks were killed humanely using CO_2_ after euthanasia of animals as described above.

### Pre- and Post-challenge monitoring

Prior to the challenge experiments, Mean HI (log_2_) titres and sero-conversion rates were analyzed as described above to determine the baseline values. Similarly, oropharyngeal swabs were collected and tested using qRT-PCR, which targets the influenza type A matrix (M) gene of A/H5N1 [40].

#### Post-challenge

Following the virus challenge, the health status of experimentally challenged birds was monitored for 10 days or more post-challenge (dpc). The parameters measured included: i) the onset of clinical signs and symptoms, ii) the duration of the clinical signs, iii) daily and cumulative mortality, and iv) the mean death time.

#### Virus shedding

Oropharyngeal swabs were collected from individual chickens at 3, 6 and 10dpc. Each swab was immediately inserted into 1 ml of viral transport medium, processed and tested separately for excretion of the challenge virus. Virus detection and quantification were conducted in a Taqman qRT-PCR, targeting the influenza type A matrix (M) gene [40]. Briefly, RNA extraction was performed according to the manufacturer’s recommendations using the QIAamp viral RNA Mini kit (Qiagen, Hilden, Germany). Genome amplification, detection and analysis were performed in a Stratagen MX3005P machine (Agilent, California, USA). An absolute quantification of the AIV matrix gene specific RNA was achieved relative to a standard curve, based on the tenfold dilution of an in vitro transcribed RNA template of the challenge virus. The current detection limit of the qRT-PCR is 2.3 copies. A cut threshold (Ct) value of 40 was selected as the cut-off between positive and negative results; therefore samples with a Ct higher than 40 were considered negative for AIV. The results were expressed as the number of M gene copies per ml of swab sample solution.

The viral load was expressed as the mean values of the M gene copies/ml per swab sample (log_10_ PCR copies) detected daily. The mean viral load shed per group was calculated only for live positive shedders per group per day. The number and proportion of shedders was calculated as the percentage of shedders of the total live birds in a group at one time point, and the level of protection against morbidity and mortality conferred by a given vaccination regime was determined as the proportion of healthy and surviving birds respectively in a group during the monitoring period after the challenge.

### Statistical analysis

Systematically collected data on parameters of interest were analyzed using descriptive and appropriate tests of the hypotheses were conducted in SPSS v 21 (IBM, 2012). The variations within and between groups were analyzed using a One-Way ANOVA and a *P*-value <0.05 was considered statistically significant for all tests.

## Supporting Information

S1 Table Weekly mean HI titres (log2 ± SD) using A/Swan/Hungary/4999/2006) rHVT/Ag that indicate the immune response to the rHVT-H5 vaccination. S1 Table legend: Different upper case letters in a row denote the presence of statistically significant (p ≤0.05) differences. *Group I (vaccinated with rHVT-H5 vaccine at 1 day old), Group II (vaccinated with inactivated KV-H5 vaccine at 8 days old), Group III (unvaccinated control).
(DOCX)

S2 Table Weekly mean HI titres (log2 ± SD) measured using (A/chicken/Egypt/Q1995D/2010) V/H5N1/Ag that indicates the immune response to the KV-H5 vaccination. S2 Table legend: Different upper case letters in a row denote the presence of statistically significant (p ≤0.05) differences. *Group 1 (vaccinated with rHVT-H5 vaccine at 1 day old), Group II (vaccinated with inactivated KV-H5 vaccine at 8 days old), Group III (unvaccinated control).
(DOCX)

S3 Table Weekly mean HI titres (log2 ± SD) measured using (A/chicken/Egypt/128S/2012) C/H5N1/Ag that indicates the immune response to the challenge virus. S3 Table legend: Different upper case letters in a row denote the presence of statistically significant (p ≤0.05) differences. *Group 1 (vaccinated with rHVT-H5 vaccine at 1 day old), Group II (vaccinated with inactivated KV-H5 vaccine at 8 days old), Group III (unvaccinated control).
(DOCX)

## Abbreviations

AI: Avian influenza
dpc: days post challenge
EID_50_: Embryo Infective Dose (50%)
HAU: Haemagglutination unit
rHVT: recombinant turkey herpes virus
MEFLUVAC: Bivalent Inactivated H5N1 vaccine
