# Supplementary material for: Protective Efficacy of Recombinant Turkey Herpes Virus (rHVT-H5) and Inactivated H5N1 Vaccines in Commercial Mulard Ducks against the Highly Pathogenic Avian Influenza (HPAI) H5N1 Clade 2.2.1 Virus
Source: PLoS One. 2016 Jun 15;11(6):e0156747. doi: 10.1371/journal.pone.0156747 (PMC4909235; doi:10.1371/journal.pone.0156747)
Supplement: S1 Table — legend: Different upper case letters in a row denote the presence of statistically significant (p ≤0.05) differences. *Group I (vaccinated with rHVT-H5 vaccine at 1 day old), Group II (vaccinated with inactivated KV-H5 vaccine at 8 days old), Group III (unvaccinated control). (DOCX) [file pone.0156747.s001.docx]

Supplementary Table 1. Weekly HI mean titres (log2 ± SD) using (A/Swan/Hungary/4999/2006) rHVT/Ag that indicate the immune response to the rHVT-H5 vaccination

| Age  (Weeks) | Group* | | |
| --- | --- | --- | --- |
|  | I | II | III |
| 0 (day 1) | 3.8±1.3^a^ | 4.1±1.1^a^ | 4.1±1.5^a^ |
| 1 | 1.9±1.6^a^ | 2.3±1.8^b^ | 2.8±1.3^c^ |
| 2 | 0.5±1.1^a^ | 0.5±1.3^a^ | 0.7±1.2^a^ |
| 3 | 1.5±1.5^a^ | 0.5±1^b^ | 0±0^c^ |
| 4 | 2±1.7^a^ | 0.4±1^b^ | 0±0^c^ |
| 5 | 2.5±1.1^a^ | 1.5±1.5^b^ | 0±0^c^ |
| 6 | 3.5±1.3^a^ | 2.1±1.2^b^ | 0±0^c^ |

*Different upper case letters in a row denote the presence of statistically significant (p ≤0.05) differences*.

**Group I (vaccinated with rHVT-H5 vaccine at 1 day old), Group II (vaccinated with inactivated KV-H5 vaccine at 8 days old), Group III (unvaccinated control).*
