# Supplementary material for: Protective Efficacy of Recombinant Turkey Herpes Virus (rHVT-H5) and Inactivated H5N1 Vaccines in Commercial Mulard Ducks against the Highly Pathogenic Avian Influenza (HPAI) H5N1 Clade 2.2.1 Virus
Source: PLoS One. 2016 Jun 15;11(6):e0156747. doi: 10.1371/journal.pone.0156747 (PMC4909235; doi:10.1371/journal.pone.0156747)
Supplement: S3 Table — legend: Different upper case letters in a row denote the presence of statistically significant (p ≤0.05) differences. *Group 1 (vaccinated with rHVT-H5 vaccine at 1 day old), Group II (vaccinated with inactivated KV-H5 vaccine at 8 days old), Group III (unvaccinated control). (DOCX) [file pone.0156747.s003.docx]

Supplementary Table 3. Weekly mean HI titres (log2 ± SD) measured using (A/chicken/Egypt/128S/2012) C/H5N1/Ag that indicate the immune response to the challenge virus

| Age  (Weeks) | Group* | | |
| --- | --- | --- | --- |
|  | I | II | III |
| 0 (day 1) | 2.9±1.3^a^ | 2.8±1.2^a^ | 3.1±1.4^a^ |
| 1 | 1.5±1.4^a^ | 1.5±1.4^a^ | 1.7±1.5^a^ |
| 2 | 0±0^a^ | 0±0^a^ | 0±0^a^ |
| 3 | 0±0^a^ | 1.7±1.3^a^ | 0±0^a^ |
| 4 | 1.1±1.3^a^ | 2.4±1.4^b^ | 0±0^c^ |
| 5 | 1.8±1.4^a^ | 2.8±1.1^b^ | 0±0^c^ |
| 6 | 2.4±1.1^a^ | 3.5±1.1^b^ | 0±0^c^ |

*Different upper case letters in a row denote the presence of statistically significant (p ≤0.05) differences*

**Group 1 (vaccinated with rHVT-H5 vaccine at 1 day old), Group II (vaccinated with inactivated KV-H5 vaccine at 8 days old), Group III (unvaccinated control).*
